# Vaccine Evolution and Its Application to Fight Modern Threats

**DOI:** 10.3389/fimmu.2019.01722

**Published:** 2019-07-25

**Authors:** Emanuele Andreano, Ugo D'Oro, Rino Rappuoli, Oretta Finco

**Affiliations:** ^1^Department of Life Sciences, University of Siena, Siena, Italy; ^2^GlaxoSmithKline, Siena, Italy; ^3^vAMRes Lab, Toscana Life Sciences, Siena, Italy; ^4^Faculty of Medicine, Imperial College, London, United Kingdom

**Keywords:** vaccines, infectious diseases, antimicrobial resistance (AMR), vaccine development, vaccinology

## Abstract

Before the development of the first vaccine, infectious diseases were a major cause of death around the globe with life expectancy estimated to be <50 years. Three measures have helped to drastically reduce the burden of infectious diseases but only vaccines have proven to be able to eradicate infectious agents. Herein, we describe new methodologies that have paved the way for what is currently known as modern vaccinology and the use of vaccines to tackle antimicrobial resistance, the biggest global threat of our time.

## The Burden of Infectious Diseases Before Antibiotics and Vaccine Intervention

Infectious diseases have always had a devastating impact on humankind. Some of the most catastrophic pandemics of our history include the Justinian plague (542-546 AD), which had a tragic toll of 100 million deaths, the bubonic plague (1347-50 AD), also known as the “Black Death,” which erased one-third of the entire human population (1, 2), and more recently the “Spanish” influenza in 1918 which caused ~50–100 million deaths worldwide reducing the European population by half (3–5). Before the introduction of effective preventive and therapeutic strategies, life expectancy was estimated to be <50 years and bacterial infections were the imperative toll setting this limit (6). This scenario changed with the introduction of three measures that helped to dramatically reduce the death burden caused by infectious diseases. The measures include hygiene, antibiotics, and vaccination (7, 8). The introduction of penicillin in 1929 (9), and its first use in humans a decade later (10), led to a dramatic reduction of mortality caused by infectious diseases. Unfortunately, in 1940 the first case of a penicillin resistant *E. coli* strain was documented and by the late 1960s over 80% of *S. aureus* strains acquired the same resistance (10–12). Therefore, despite the use of antibiotics resulted to be an outstanding first line of defense to treat infections, pathogens have shown to quickly acquire resistance phenotypes after only few years from their introduction (13). Vaccines, on the other hand, have only rarely shown to induce resistant phenotypes as they usually aim to elicit a multi-targets immune response and their prophylactic use reduces the likelihood of spreading resistant-conferring mutations (14). Indeed the smallpox vaccine introduced in 1796, and subsequently manufactured from infected calf skin (15), has led to the eradication of this infectious agent in 1988 (16, 17). Therefore, despite the fact that antibiotics and vaccines are pivotal interventions against infectious diseases, vaccination has been the sole intervention capable of eradicating an infectious agent and, given its potential, it can also be considered as the most appropriate solution against future global threats represented by infectious diseases (18–20).

## Reverse Vaccinology and the Development of Modern Vaccines

Since Edward Jenner first vaccinated an 8 year old boy in 1796 by inoculating fresh cowpox lesion matter (21), enormous leaps forward have been made in the field of vaccine development. Empirical approaches like attenuation and inactivation of microorganisms were the first steps forward to modern vaccinology (22). Recently, new technologies such as glycoconjugates and the introduction of novel vaccine adjuvants changed the field of vaccines, however the biggest change came with the first sequencing of the *Heamophilus influenzae* whole genome in 1995, a discovery that allowed the birth of “Reverse Vaccinology,” a genome-based approach to vaccine development (23, 24). This approach, following the sequencing and analysis of the *Neisseria meningitidis* serogroup B strain whole genome, allowed the identification of novel candidates and the development of a four-component meningococcus B vaccine (4CMenB) (25, 26). This recently licensed vaccine has already shown incredible effectiveness in the UK with 82.9% protection against all MenB strains in infants (27). The evolution of vaccine development further moved forward with the advancement of new methodologies and technological breakthroughs. Indeed, in 2016 the “reverse vaccinology 2.0” entered the stage. With this approach, the human immune system is analyzed at a single cell level allowing the characterization of the antibody response like never before (28). The gain of knowledge acquired by this approach allows to rapidly identify highly immunogenic antigens to develop novel and more efficacious vaccine candidates. The RSV fusion protein (F) case is a major example of the phenomenal power of the reverse vaccinology 2.0. Indeed, human B cells were directly isolated from RSV convalescent donors and cultured to naturally produce human monoclonal antibodies (humAbs). Among all the antibodies screened for RSV neutralization *in vitro*, the humAbs named D25 resulted in the most potent antibody with a median half-maximal inhibitory concentration (IC50) of 2.1 ng/ml (100–150 times more than palivizumab, the only monoclonal antibody approved by the FDA for RSV prevention in infants) (29). Interestingly, D25 was not capable of binding to the RSV F-protein in its post-fusion conformation, the only vaccine candidate available at the time against RSV (30). Then, McLellan and coworkers had the brilliant intuition to test D25 complex with the RSV F-protein to perform structural studies. This experiment was paramount in solving the crystal structure of RSV F-protein in its pre-fusion conformation (preF) which in turn led to the design of a stabilized RSV preF molecule (30, 31). Following the production of a soluble preF reagent, numerous human neutralizing antibodies have been identified allowing a deep characterization of the antigen surface and the identification of two preF-specific antigenic sites that have shown incredible high neutralization potency (32). The effectiveness of the preF antigen has already been proven in different animal models (mice, rhesus macaques, and calves) further supporting the potential of RSV preF as an ideal vaccine candidate against this pneumovirus (10, 13). The power of reverse vaccinology 2.0 has allowed, in <5 years since preF stabilization, to start clinical trials that are currently on-going to develop the first vaccine against RSV (7). This approach, which has found broad applicability to fight viral infections, could also be considered as a key stratagem to tackle bacterial infections.

## Use of Peptide-Antigen Derived for Germline Targeting Vaccinology

The production of germline-targeting (GT) antigens for vaccine development is another pivotal example that underlies the outstanding potential of reverse vaccinology 2.0. Indeed, the combined knowledge acquired by the identification and characterization of novel antigens plus the functional/genetic analysis of human monoclonal antibodies naturally produced by infected or vaccinated human donors, can be used to design antigen-derived peptides, capable of tailoring the antibody immune response. In case of highly variable pathogens such as HIV, the use of the whole antigen can result in a strain specific response, while the development of GT-antigens can lead to the elicitation of broadly neutralizing antibodies (bnAbs) capable of clearing multiple infective strains. This is a two-step approach which, using different rationally designed immunogens, aims to: (1) prime the germline precursor B cell of antibodies previously shown to possess broadly neutralizing activity; (2) shepherding the bnAb population by driving their maturation affinity toward the highly immunogenic epitope of interest. GT-vaccinology has been used to elicit a specific class of HIV-1 gp120 CD4-binding site specific-bnAbs known as VRC01, through the use of engineered outer domain germline-targeting (eOD-GT) peptides (33). The interest to prime VRC01-bnAbs arises from their ability to mimic the CD4-binding to the gp120 receptor binding site and their capability to potently neutralize (median IC50 40 ng/mL) up to 98% of a large panel of global HIV-1 isolates (34, 35). An in-depth analysis of the VRC01 genetic features has shown peculiarities in this class of bnAbs. They classically derive from an extensively mutated (32–48%) VH1-2^*^02 heavy chain germline which pairs with light chains, presenting a rare five amino acid long CDR3 motif (usually QQYEF) (36). These analyses were paramount for the development of novel and potentially therapeutic candidates to fight HIV infections. Examples of the use of VRC01-bnAbs as a therapeutic tool are the monoclonal antibodies named VRC-HIVMAB060-00-AB (VRC01) and a FC-modified version of this latter named VRC01LS. These two bnAbs are currently under clinical investigation (NCT02568215, NCT02716675, and NCT02599896) evaluating safety and efficacy in reducing acquisition of HIV-1 infection (37–40). In addition to monoclonal antibody development and application, the knowledge acquired from these studies and the ability to selectively expand this class of bnAbs upon immunization (41), have allowed the development of specific peptides as vaccine candidates capable of shepherding the immune system toward a VRC01-like antibody response. The most promising candidate is the tailored immunogen named eOD-GT8 60-subunit self-assembling nanoparticle (eOD-GT8 60mer) (36, 42) which has shown superior affinity and breadth of binding to germline-reverted VRC01-like bnAbs (41).

The HIV case described above further confirms the outstanding power of reverse vaccinology 2.0. Indeed, in only 3 years since its design and stabilization (43), the eOD-GT8 60mer antigen is under investigation in a phase I clinical trial in healthy adults aimed at assessing safety, tolerability and immunogenicity of this germline-targeting immunogen (NCT03547245).

## Vaccines for the Future: the Fight Against Antimicrobial Resistance

**Figure 1:**
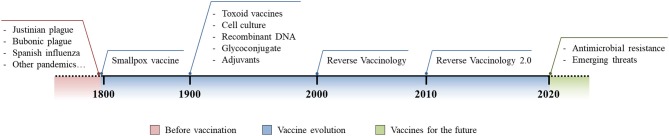
Vaccine evolution. Schematic representation of the burden of infectious diseases before vaccination was introduced (red), technological and methodological advances in vaccinology following the introduction of the first vaccine (blue), and the future use and implementation of vaccine development to fight modern threats (green).

Despite antibiotics being the only lifesaving tool in fighting acute bacterial infection, as Stanley Falkow said (3), they are creating some problems of their own. In fact, the improper and excessive use of antibiotics has pressured bacteria to acquire antibiotic resistant phenotypes and this problem is currently growing out of control. Bacteria have shown several mechanisms to acquire antibiotic resistance and examples include the expression of β-lactamases, efflux pumps, modification of the cellular surface, and gene mutations to alter those molecules that are targeted by antibiotics (4). This phenomenon, known as antimicrobial resistance (AMR), is arguably one of the biggest threats that our world is facing today. Indeed, up to 700,000 deaths each year are AMR-related and these have been estimated to increase up to 10 million by 2050, exceeding the 8.2 million deaths per year caused by cancer today (8, 44). A solution to this alarming threat would be the prevention of antibiotic resistant bacteria infections through vaccination, a strategy that has already proven its great value to humanity (6). Several reasons suggest that vaccines would be a promising solution against AMR. First, antibiotics have shown to rapidly become obsolete and resistance emerges soon after their introduction, while vaccines allow long-lasting protection against infections and resistance has only rarely evolved after vaccination (13). Second, while antibiotics only hit a few metabolic target vaccines, based on the selected strategy, they can elicit a broad multi-target immune response reducing the probability of the evolution of resistant mutations. Furthermore, although major investments have been made to enrich antibiotic R&D pipelines, the discovery of innovative antimicrobial targets are running dry since the 1970s. Therefore, given the incredibly high pace with which pathogens are capable of developing resistance to new classes of antibiotics, focusing our attention exclusively on antibiotic R&D will not be sufficient (13, 45). In a marked contrast, thanks to incredible technological advancements of the last few decades, vaccine R&D pipelines are promising for the development of innovative and highly effective vaccines which can have an important contribution in controlling AMR (13, 18). Finally, antibiotics can only be used to treat individuals already infected, while successful vaccination campaigns can prevent the occurrence of infection, reducing the spread of the infectious agent and protecting the whole population through herd immunity (8, 20, 46). Vaccine evolution has allowed us to address several unmet medical needs and, given all of the reasons stated above, it should be considered a key solution in fighting emerging threats such as AMR (Figure 1).

## Conclusions

Since their introduction, vaccines have helped save billions of lives all over the world. Empirical approaches were not sufficient to support the development of vaccines against pathogens for which no preventive strategies or treatments were available. Methodological and technological advancements have introduced the world to modern vaccinology approaches which have unlocked the possibility to develop novel vaccines against virtually any pathogen. The RSV and HIV case studies reported herein, are clear examples of how innovative technologies and their corollary applications have paved the way for new experimental approaches capable of tackling and possibly addressing these unmet global medical needs. Vaccines have provided the basis for a global and sustainable public health in the past and they can potentially continue to do so by addressing major and upcoming global threats like AMR.

## Author Contributions

All authors listed have made a substantial, direct and intellectual contribution to the work, and approved it for publication.

### Conflict of Interest Statement

RR, OF, and UD'O are full-time employees of GSK group of companies. EA participated in a postgraduate program at GSK.
